# Novel insights into immune checkpoints in HIV/SHIV infection: from SHIV_SF162P3_-infected elite controllers to therapeutic strategy

**DOI:** 10.1128/jvi.00785-25

**Published:** 2025-07-10

**Authors:** Yuting Sun, Chenbo Yang, Peiwen Liu, Zhe Cong, Jiahui Luo, Ling Tong, Jingjing Zhang, Jiahan Lu, Ziqing Jia, Lin Zhu, Qiuhan Lu, Ting Chen, Qiang Wei, Dan Li, Rui Hou, Jing Xue

**Affiliations:** 1 NHC Key Laboratory of Human Disease Comparative Medicine, Beijing Key Laboratory for Animal Models of Emerging and Remerging Infectious Diseases, Institute of Laboratory Animal Science, Chinese Academy of Medical Sciences and Peking Union Medical College 12501, Beijing, China; 2 Harry Perkins Institute of Medical Research, QEII Medical Centre and Centre for Medical Research, the University of Western Australia 569352, Perth, Western Australia, Australia; 3 National Center of Technology Innovation for animal model, National Human Diseases Animal Model Resource Center, Institute of Laboratory Animal Science, Chinese Academy of Medical Sciences 71046, Beijing, China; 4 Center for AIDS Research, Chinese Academy of Medical Sciences and Peking Union Medical College 12501, Beijing, China; 5 State Key Laboratory of Respiratory Health and Multimorbidity, Key Laboratory of Pathogen Infection Prevention and Control (Peking Union Medical College), Ministry of Education, Institute of Laboratory Animal Science, Chinese Academy of Medical Sciences 71046, Beijing, China; 6 National Key Laboratory of Intelligent Tracking and Forecasting for Infectious Diseases, National Center for AIDS/ STD Control and Prevention, Chinese Center for Disease Control and Prevention 12415, Beijing, China; 7 Shenzhen Clinical Research Centre for Geriatrics, Shenzhen People’s Hospital (The Second Clinical Medical College, Jinan University, The First Affiliated Hospital, Southern University of Science and Technology) Shenzhen, Guangdong, China; Icahn School of Medicine at Mount Sinai, New York, New York, USA

**Keywords:** immune checkpoints, HIV/SHIV, elite controllers, TIGIT, non-human primates

## Abstract

During chronic HIV-1 infection, the upregulation of immune checkpoints (ICs) on lymphocytes contributes to persistent immune dysfunction, T-cell exhaustion, and inadequate virological control, highlighting the potential role of ICs in HIV functional cure strategy. Despite extensive research on ICs, the expression dynamics and immunological impact of ICs in natural HIV/SHIV infection, particularly among antiretroviral therapy (ART)-free elite controllers (ECs), remain poorly understood. In this study, we monitored a cohort of SHIV_SF162P3_-infected rhesus macaques (RMs), identifying four ECs and four progressors (PGs) through longitudinal evaluation. We observed low-level expression of ICs in both peripheral blood and lymph nodes of ECs, characterized by a particularly pronounced restriction in the expression of TIGIT and BTLA. This attenuated IC profile correlated with enhanced T-cell functionality, reduced exhaustion markers, and reduced viral reservoirs in peripheral and lymphoid tissues. Transcriptomic profiling revealed that TIGIT is a critical checkpoint marker involved in multiple synergistic cofunctions related to HIV/SIV-specific immune regulation. Collectively, our findings establish a dual role for ICs in perpetuating T-cell exhaustion and viral reservoir persistence, paving the way for IC blockade in future HIV cure strategies.

**IMPORTANCE:**

Rhesus macaques spontaneously controlling simian-human immunodeficiency virus (SHIV) without antiretroviral therapy have low-level expression of immune molecules (ICs), characterized by TIGIT and BTLA. These molecules are linked to enhanced immune function and reduced viral presence in peripheral blood and lymph nodes. Transcriptomic profiling revealed that TIGIT is a critical checkpoint marker involved in multiple synergistic cofunctions related to HIV/SIV-specific immune regulation in both humans and macaques. Blocking TIGIT improved polyfunctional T-cell responses, thereby offering a potential new treatment strategy and providing critical insights for developing a functional HIV cure.

## INTRODUCTION

The ability of HIV and its nonhuman primate counterpart, SIV, to establish an early viral reservoir during acute infection, coupled with the anergy of the immune system to suppress ongoing viral replication, results in a lifelong persistence of HIV/SIV in affected individuals, rendering them incurable. Most people living with HIV experience rapid viral proliferation and a progressive decline in CD4^+^ T-cell counts in the absence of highly active antiretroviral therapy (ART), a condition commonly termed progressors (PGs). On the contrary, a unique and rare subgroup of people living with HIV, now known as elite controllers (ECs), accounts for less than 1% of people living with HIV (1–3). These individuals can maintain undetectable plasma HIV-1 RNA levels and stable CD4^+^ T-cell counts without ART. Although the specific mechanisms underlying natural virological control remain inadequately understood and warrant further investigation, the potential for virological control observed in ECs could be utilized as an ideal model for elucidating the interplay between host and viral factors, potentially revealing pathways critical for achieving an HIV-1 functional cure.

Recent studies have highlighted the involvement of HIV-1 proviruses and reservoirs (4–7), host protective alleles (7), and robust HIV-specific immune responses (2, 8, 9) in the mechanisms of virological control (10). IC systems, including PD-1, CTLA-4, TIGIT, LAG-3, TIM-3, and CD160, play a pivotal regulatory role in HIV-1 disease progression (11–13). The expression of ICs on CD4^+^ and CD8^+^ T cells is generally positively correlated with viral loads and inversely correlated with the CD4^+^/CD8^+^ T-cell ratio. Upregulation of ICs enhances coinhibitory signaling, which impairs the proliferation and cytokine production of HIV-specific CD4^+^ and CD8^+^ T cells (13). Furthermore, increased IC expression contributes to the enrichment of HIV reservoirs and latency establishment, with CD4^+^ T cells expressing PD-1, TIM-3, TIGIT, and LAG-3 representing key cellular HIV reservoirs (14–20). Encouraging findings suggest that blockade of ICs can elicit therapeutic benefits by reinvigorating exhausted T cells and reversing HIV-1 latency in both preclinical and clinical studies (21). Notably, combination therapies incorporating immune checkpoint blockade (ICB) with other immunomodulators, such as PD-1 inhibitors combined with anti-IL10, have shown promise in achieving durable control of viral rebound (22, 23). However, further investigation into systematic IC dynamics during long-term natural infection is necessary to address existing knowledge gaps, particularly in elucidating regional immunity in secondary lymphoid organs and the direct immune restoration and virological control mediated by ICB immunotherapy.

To systematically investigate the mechanisms governing dysregulated IC expression during natural infection, we established a longitudinally monitored cohort of SHIV_SF162P3_-infected rhesus macaques (RMs). PGs and ECs were defined through rigorous longitudinal monitoring of CD4^+^ T-cell counts and viral loads, complemented by cross-species RNA sequencing (RNA-seq) validation of compelling IC expression profiles between humans and RMs, providing a novel animal model for investigating systemic host immunological response and viral factors in sustained virological control. We subsequently conducted comprehensive analyses of dynamic IC expression patterns and functional signatures on circulating and lymph node (LN)-derived T lymphocytes across PGs and ECs, aiming to elucidate the immunomodulatory mechanisms that sustain virological controllers and guide the optimization of targeted HIV remission strategies.

In this study, we systematically monitored the expression of total ICs, including PD-1, CTLA-4, LAG-3, CD160, TIGIT, and BTLA, on resting CD4^+^ and CD8^+^ T cells in both PGs and ECs during viral infection. Our findings indicate that ECs exhibited lower TIGIT and BTLA expressions on resting CD4^+^ T-cell subsets in peripheral blood and LNs, which was associated with reduced viral DNA burden and enhanced SIV-specific CD8^+^ T-cell response. Further transcriptomic analysis revealed TIGIT’s involvement in immune exhaustion and regulation pathways, confirming its role as a key checkpoint marker. Collectively, the immunologic and virologic effects and therapeutic potential of ICs in HIV/SHIV infection demonstrate their potential as therapeutic targets for HIV functional cure.

## RESULTS

### PGs and ECs with specific MHC class I alleles established from the 47 SHIV_SF162P3_-infected RMs cohort

**Fig 1:**
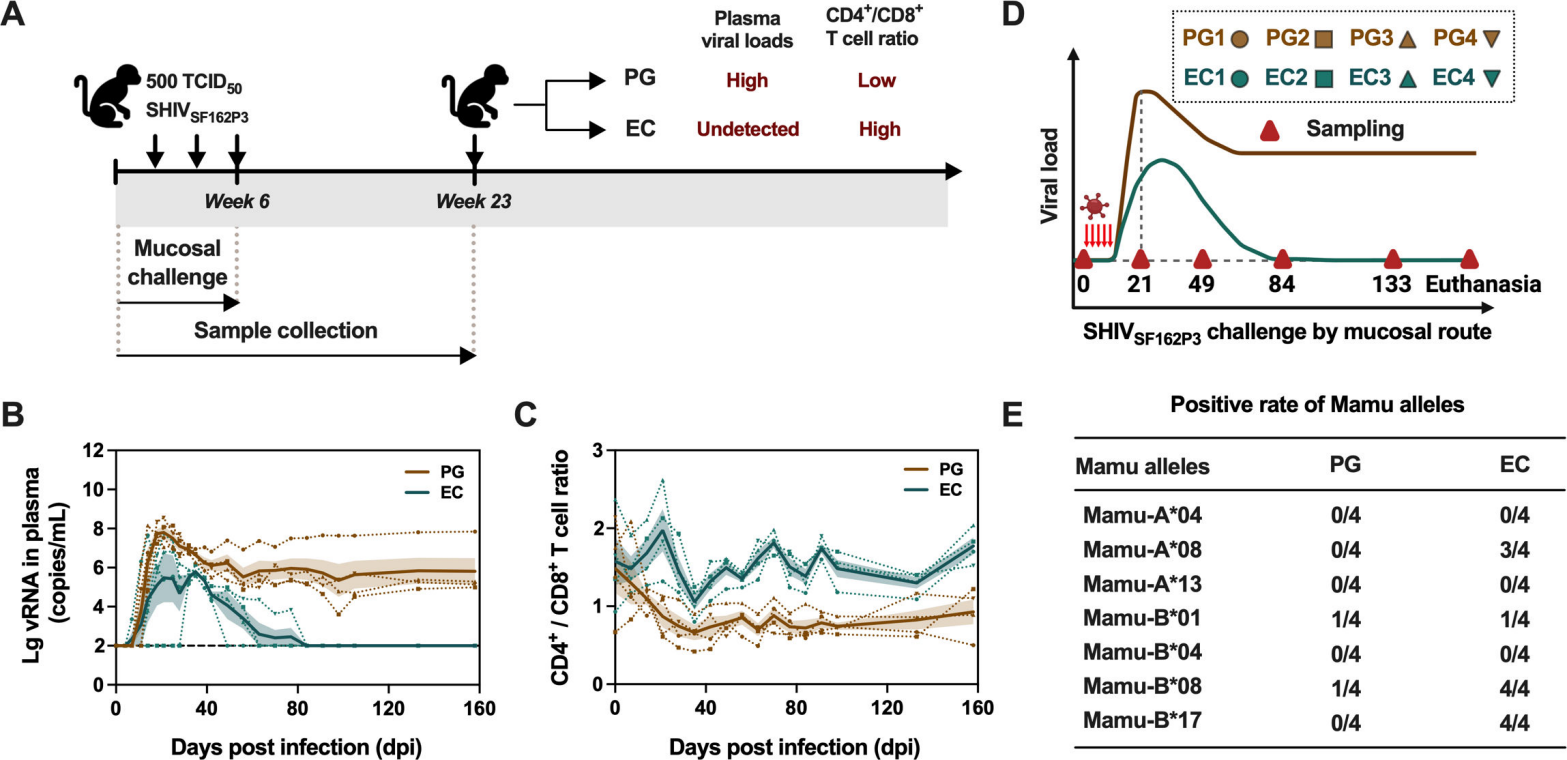
Study design and the classification of PGs and ECs. (**A**) Study design. Forty-seven RMs were challenged with 500 TCID_50_ of SHIV_SF162P3_ via rectal or vaginal routes bi-weekly over a 6-week period. Plasma viral loads and CD4^+^/CD8^+^ T-cell ratios were continuously monitored for 17 weeks post-exposure. Four out of forty-seven RMs with undetected plasma virus levels and high CD4^+^/CD8^+^ T-cell ratios were classified as ECs, while another four RMs with high plasma viral loads and low CD4^+^/CD8^+^ T-cell ratios were classified as PGs. (**B and C**) Plasma viral loads (**B**) and CD4^+^/CD8^+^ T-cell ratios (**C**) of PGs (yellow) and ECs (green). (**D**) Schematic illustration of RM models for PGs and ECs by repeated mucosal challenge. Peripheral blood was collected from the indicated time points (represented by red triangles) for the measurement of ICs expression on CD4^+^ and CD8^+^ T-cell subsets in the following studies. (**E**) The comparison of Mamu alleles between PGs and ECs.

To establish a cohort of RMs consisting of PGs and ECs to characterize their IC expression profiles, we conducted a study involving 47 RMs from a previous study. All animals were subjected to biweekly challenges with 500 TCID_50_ of SHIV_SF162P3_ via mucosal routes (rectal or vaginal) over a 6-week period (Fig. 1A). Plasma viral loads and CD4^+^/CD8^+^ T-cell ratios were systematically monitored for a duration of 17 weeks. Notably, 4 out of the 47 RMs (8.5%) (24, 25) maintained undetectable viral loads during the chronic phase and exhibited a relatively high and stable CD4^+^/CD8^+^ T-cell ratios without therapeutic intervention (Fig. 1B to 1D). To further elucidate the immunological status of these 4 RMs, we analyzed their MHC class I alleles and identified three alleles, including Mamu-A*08, Mamu-B*08, and Mamu-B*17, which were present at elevated frequencies in all 4 RMs, associated with reduced viral loads and sustained CD4^+^/CD8^+^ T-cell ratios, a characteristic notably observed in RMs that achieve complete viral suppression, commonly referred to as ECs (24, 25) (Fig. 1E). By contrast, 4 RMs exhibited persistent high-level viremia (>10^5^ copies/mL), and progressive depletion of CD4^+^ T cells were categorized as PGs.

### Low-level expression of BTLA and TIGIT in circulating T cells in ECs

To investigate the potential role of ICs in the progression of SHIV disease, we conducted a quantitative analysis of IC expression profiles and cellular responses in both PGs and ECs. Peripheral blood samples were collected at five timepoints stratified into three phases: baseline (Day 0 before infection), the acute infection phase (21 days and 49 days post-infection), and the chronic infection phase (84 days and 133 days post-infection) (Fig. 1D).

**Fig 2:**
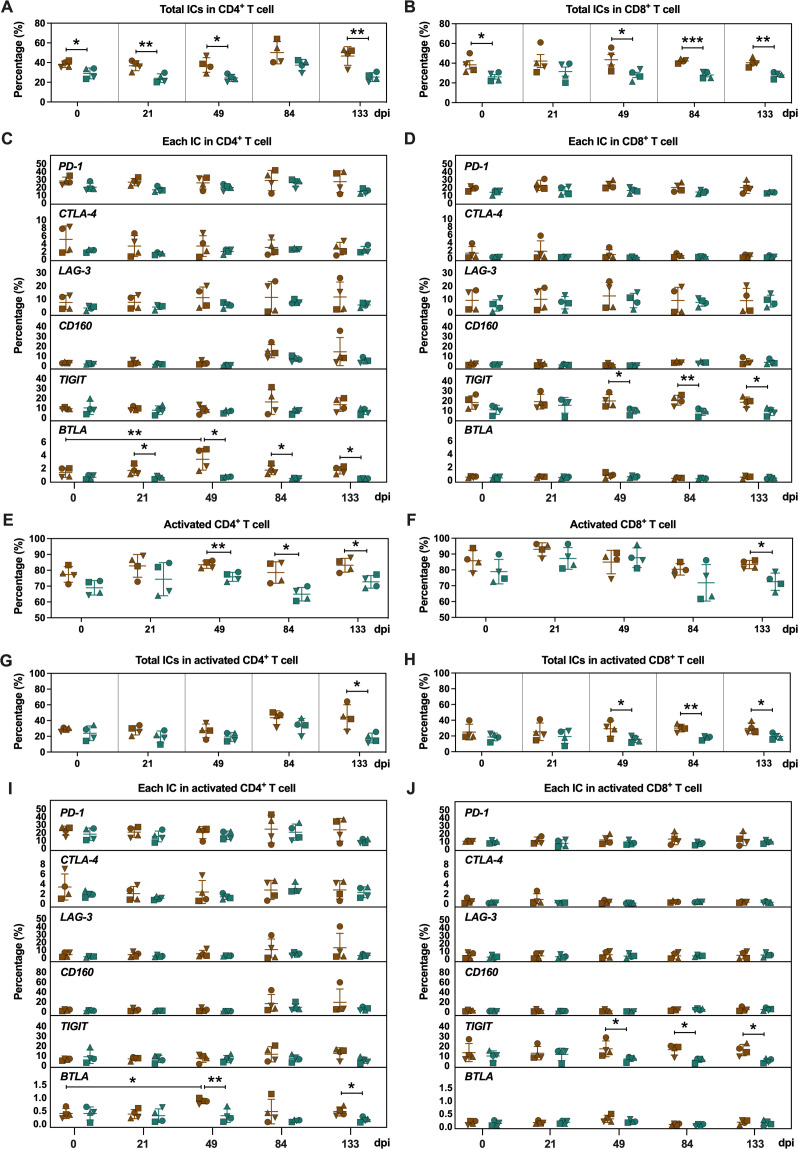
The expression of ICs on peripheral CD4^+^ and CD8^+^ T-cell subsets of PGs and ECs. (**A and B**) Comparison of the frequencies of total ICs^+^ CD4^+^ T cells (**A**) and CD8^+^ T cells (**B**) from PBMCs between PGs and ECs. (**C and D**) Comparison of the frequencies of individual IC^+^ CD4^+^ T cells (**C**) and CD8^+^ T cells (**D**) from PBMCs between PGs and ECs. (**E and F**) The expression of HLA-DR and CD38 on CD4^+^ T cells (**E**) and CD8^+^ T cells (**F**) among PGs and ECs. (**G and H**) Comparison of the frequencies of total ICs^+^-activated (HLA-DR^+^/CD38^+^) CD4^+^ T cells (**G**) and activated CD8^+^ T cells (**H**) from PBMCs between PGs and ECs. (**I and J**) Comparison of the frequencies of individual IC^+^ activated (HLA-DR^+^/CD38^+^) CD4^+^ T cells (**I**) and activated CD8^+^ T cells (**J**) from PBMCs between PGs and ECs. Statistical analyses were performed by two-tailed unpaired Student’s or Welch’s *t*-test between two groups at the indicated time points. Repeated measures ANOVA was used to compare each group over all indicated time points. Error bars represent the mean ± SD. *P < 0.05, **P < 0.01, ***P < 0.001.

We analyzed the ICs on CD4^+^ and CD8^+^ T cells at five longitudinal time points, revealing significantly lower frequencies of total ICs^+^ in CD4^+^ (0, 21, 49, and 133 days post-infection) and CD8^+^ T cells (0, 49, 84, and 133 days post-infection) in ECs across all phases (Fig. 2A and B; Fig. S1). Specifically, ECs demonstrated significantly low-level BTLA expression in CD4^+^ T cells (21, 49, 84, and 133 days post-infection), as well as diminished TIGIT expression in CD8^+^ T cells (49, 84, and 133 days post-infection) compared to PGs (Fig. 2C and D).

During the various phases of infection, ECs demonstrated a markedly low-level expression of HLA-DR and CD38, considered as the markers of T-cell activation in CD4^+^ T cells from the acute to chronic infection phase (49, 84, and 133 days post-infection), which was also observed in CD8^+^ T cells specifically during the chronic infection phase (133 days post-infection, Fig. 2E and F). Furthermore, total ICs exhibited significantly lower levels on activated (HLA-DR^+^/CD38^+^) CD4^+^ T cells during the chronic phase (133 days post-infection) and on activated CD8^+^ T cells during the acute and chronic infection phases (49, 84, and 133 days post-infection) in ECs (Fig. 2G and H). Notably, ECs exhibited significantly lower BTLA expression in activated CD4^+^ T cells (49 and 133 days post-infection, Fig. 2I) and TIGIT expression in activated CD8^+^ T cells (49, 84, and 133 days post-infection) (Fig. 2J). These data collectively delineate a T-cell phenotype specific to ECs, characterized by a diminished state of activation and restricted expression of ICs in the peripheral activated CD4^+^ and CD8^+^ T cells.

**Fig 3:**
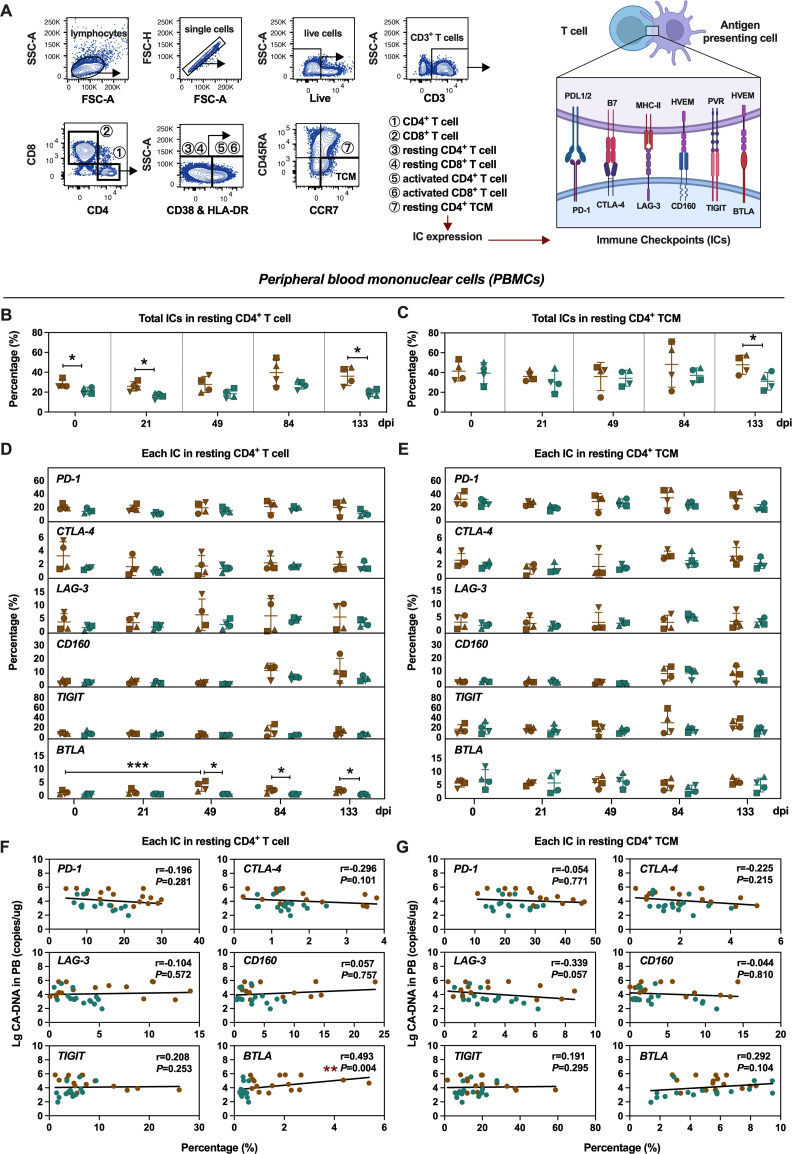
Viral DNA associated with frequencies of BTLA^+^ resting CD4^+^ T cells from PBMCs in PGs and ECs. (**A**) Schematic illustration of ICs expression measurement on CD4^+^ T cells (CD3^+^CD4^+^ T cells), CD8^+^ T cells (CD3^+^CD8^+^ T cells), resting CD4^+^ T cells (HLA-DR^-^CD38^-^CD3^+^CD4^+^ T cells), resting CD8^+^ T cells (HLA-DR^-^CD38^-^CD3^+^CD8^+^ T cells), activated CD4^+^ T cells (HLA-DR^+^/CD38^+^CD3^+^CD4^+^ T cells), activated CD8^+^ T cells (HLA-DR^+^/CD38^+^CD3^+^CD8^+^ T cells), resting central memory CD4^+^ T cells (resting CD4^+^ TCM, HLA-DR^-^CD38^-^CCR7^+^CD45RA^-^CD3^+^CD4^+^ T cells). (**B and C**) Comparison of the frequencies of total ICs^+^ resting CD4^+^ T cells (**B**) and resting CD4^+^ TCM (**C**) from PBMCs between PGs and ECs at the five indicated time points post-infection. (**D and E**) Comparison of the frequencies of individual IC^+^ resting CD4^+^ T cells (**D**) and resting CD4^+^ TCM (**E**) from PBMCs between PGs and ECs. (**F and G**) Correlation analysis of the frequencies of individual IC^+^ resting CD4^+^ T cells (**F**) and resting CD4^+^ TCM (**G**) with total proviral DNA in PBMCs across 0, 21, 49, 84, and 133 days post-infection. Statistics were performed by two-tailed unpaired Student’s or Welch’s *t*-test between two groups at the indicated time points. Repeated measures ANOVA was used to compare each group over all indicated time points. Error bars represent the mean ± SD. The Spearman non-parametric test was calculated for correlation analysis. *P < 0.05, **P < 0.01.

Next, we systematically analyzed the expression of ICs in resting CD4^+^ T cells and resting central memory CD4^+^ T cells (resting CD4^+^ TCM) from peripheral blood. Comparative analysis between PGs and ECs (Fig. 3A) demonstrated that lower expression of total ICs in resting CD4^+^ T cells was observed during all phases in ECs (0, 21, and 133 days post-infection, Fig. 3B), which was also observed in resting CD4^+^ TCM but only during the chronic infection phase (133 days post-infection, Fig. 3C).

Specifically, we observed lower BTLA expression on resting CD4^+^ T cells during both acute and chronic infection phases in ECs (49, 84, and 133 days post-infection, Fig. 3D) while maintaining comparable IC expression levels on resting CD4^+^ TCM relative to PGs (Fig. 3E). Of note, a positive correlation was observed between the frequency of BTLA in resting CD4^+^ T cells and proviral DNA levels in PBMCs (*P* < 0.01, Fig. 3F), whereas such a correlation was absent in resting CD4^+^ TCM cells (Fig. 3G).

### Proviral DNA in inguinal and mesenteric lymph nodes was positively associated with BTLA and TIGIT

**Fig 4:**
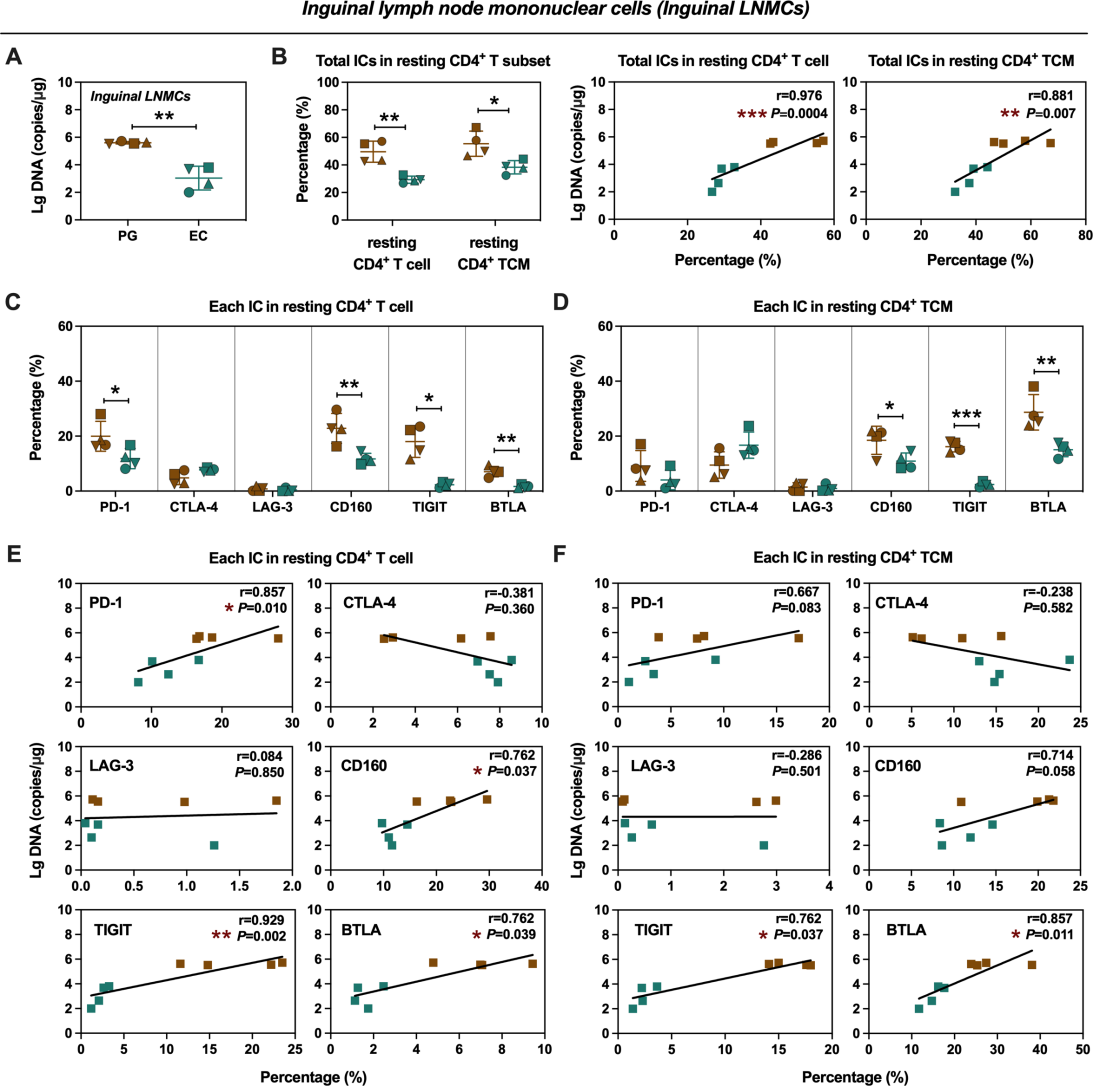
Frequencies of ICs^+^ resting CD4^+^ T-cell subsets and proviral DNA level from inguinal LNMCs in PGs and ECs. (**A**) Quantitative comparison of proviral DNA in inguinal LNMCs between PGs and ECs. (**B**) Comparison of the frequencies of total ICs^+^ resting CD4^+^ T cells and resting CD4^+^ TCM in inguinal LNMCs between PGs and ECs (left panels), and correlation analysis of the frequencies of total ICs^+^ resting CD4^+^ T cells and resting CD4^+^ TCM with proviral DNA in inguinal LNMCs (middle and right panels). (**C and D**) Comparison of the frequencies of individual IC^+^ resting CD4^+^ T cells (**C**) and resting CD4^+^ TCM (**D**) in inguinal LNMCs between PGs and ECs. (**E and F**) Correlation of the frequencies of each IC^+^ resting CD4^+^ T cells (**E**) and resting CD4^+^ TCM (**F**) with proviral DNA in inguinal LNMCs was accordingly analyzed. Statistics were performed by two-tailed unpaired Student’s or Welch’s *t*-test. Error bars represent the mean ± SD. The Spearman non-parametric test was calculated for correlation analysis. *P < 0.05, **P < 0.01, ***P < 0.001.

To investigate the relationship between ICs and proviral DNA, we assessed ICs expression in resting CD4^+^ T cells and quantified proviral DNA in both inguinal and mesenteric lymph node mononuclear cells (LNMCs) at necropsy. We found ECs harbored significantly lower levels of proviral DNA in inguinal LNMCs (Fig. 4A) and exhibited low-level expression of total ICs in resting CD4^+^ T cells and resting CD4^+^ TCM (Fig. 4B), which also showed positive correlation with proviral DNA levels (Fig. 4B). In addition, ECs exhibited broadly diminished IC expression in inguinal LNMCs, characterized by lower levels of PD-1, CD160, TIGIT, and BTLA in resting CD4^+^ T cells, as well as lower levels of CD160, TIGIT, and BTLA in resting CD4^+^ TCM compared to PGs (Fig. 4C and D). Specifically, the expression levels of PD-1, CD160, TIGIT, and BTLA exhibited a significant positive correlation with proviral DNA in resting CD4^+^ T cells (Fig. 4E). Notably, only TIGIT and BTLA expression, but not other ICs, showed a significant positive correlation with proviral DNA in resting CD4^+^ TCM (Fig. 4F).

**Fig 5:**
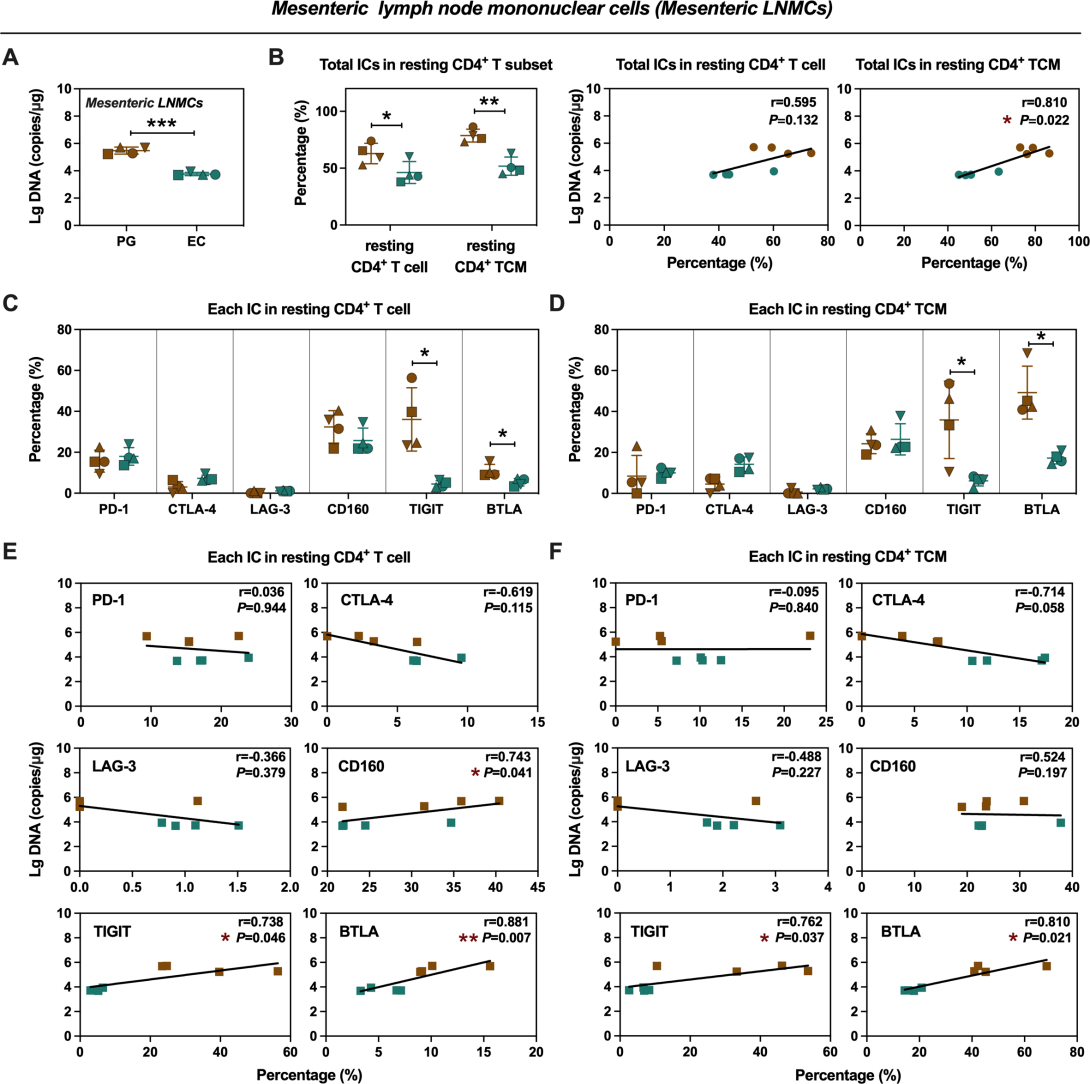
Frequencies of ICs^+^ resting CD4^+^ T-cell subsets and proviral DNA levels from mesenteric LNMCs in PGs and ECs. (**A**) Quantitative comparison of proviral DNA in mesenteric LNMCs between PGs and ECs. (**B**) Comparison of the frequencies of total ICs^+^ resting CD4^+^ T cells and resting CD4^+^ TCM in mesenteric LNMCs between PGs and ECs (left panels), and correlation analysis of the frequencies of total ICs^+^ resting CD4^+^ T cells and resting CD4^+^ TCM with proviral DNA in mesenteric LNMCs (middle and right panels). (**C and D**) Comparison of the frequencies of individual IC^+^ resting CD4^+^ T cells (**C**) and resting CD4^+^ TCM (**D**) in mesenteric LNMCs between PGs and ECs. (**E and F**) Correlation of the frequencies of each IC^+^ resting CD4^+^ T cells (**E**) and resting CD4^+^ TCM (**F**) with proviral DNA in mesenteric LNMCs was accordingly analyzed. Statistics were performed by two-tailed unpaired Student’s or Welch’s *t*-test. Error bars represent the mean ± SD. The Spearman non-parametric test was calculated for correlation analysis. *P < 0.05, **P < 0.01, ***P < 0.001.

Consistent with inguinal LNMCs’ findings, mesenteric LNMCs in ECs also exhibited significantly lower levels of proviral DNA compared to PGs (Fig. 5A). The frequency of total ICs^+^ resting CD4^+^ T cells and resting CD4^+^ TCM was significantly lower in ECs, with total ICs expression in resting CD4^+^ TCM positively correlating with proviral DNA levels in mesenteric LNMCs (Fig. 5B). Remarkably, low-level TIGIT and BTLA expressions were observed in both resting CD4^+^ T cells and resting CD4^+^ TCM in mesenteric LNMCs from ECs (Fig. 5C and D). Notably, TIGIT and BTLA expression in both resting CD4^+^ T cells and resting CD4^+^ TCM demonstrated a strong positive correlation with proviral DNA levels (Fig. 5E and F). By contrast, other ICs showed no such association, except for a slight correlation observed with CD160 in resting CD4^+^ T cells (Fig. 5E). Collectively, ECs exhibited generally low-level expression levels of ICs in both inguinal and mesenteric LNMCs, with a notable emphasis on TIGIT and BTLA. In addition, there was a significant positive correlation observed between the expression of TIGIT and BTLA and the presence of proviral reservoirs, suggesting potential mechanisms that may contribute to viral control in ECs.

### Transcriptomic analysis reveals low-level expressions of Tim-3, TIGIT, and LAG-3 in ECs of RMs and humans

**Fig 6:**
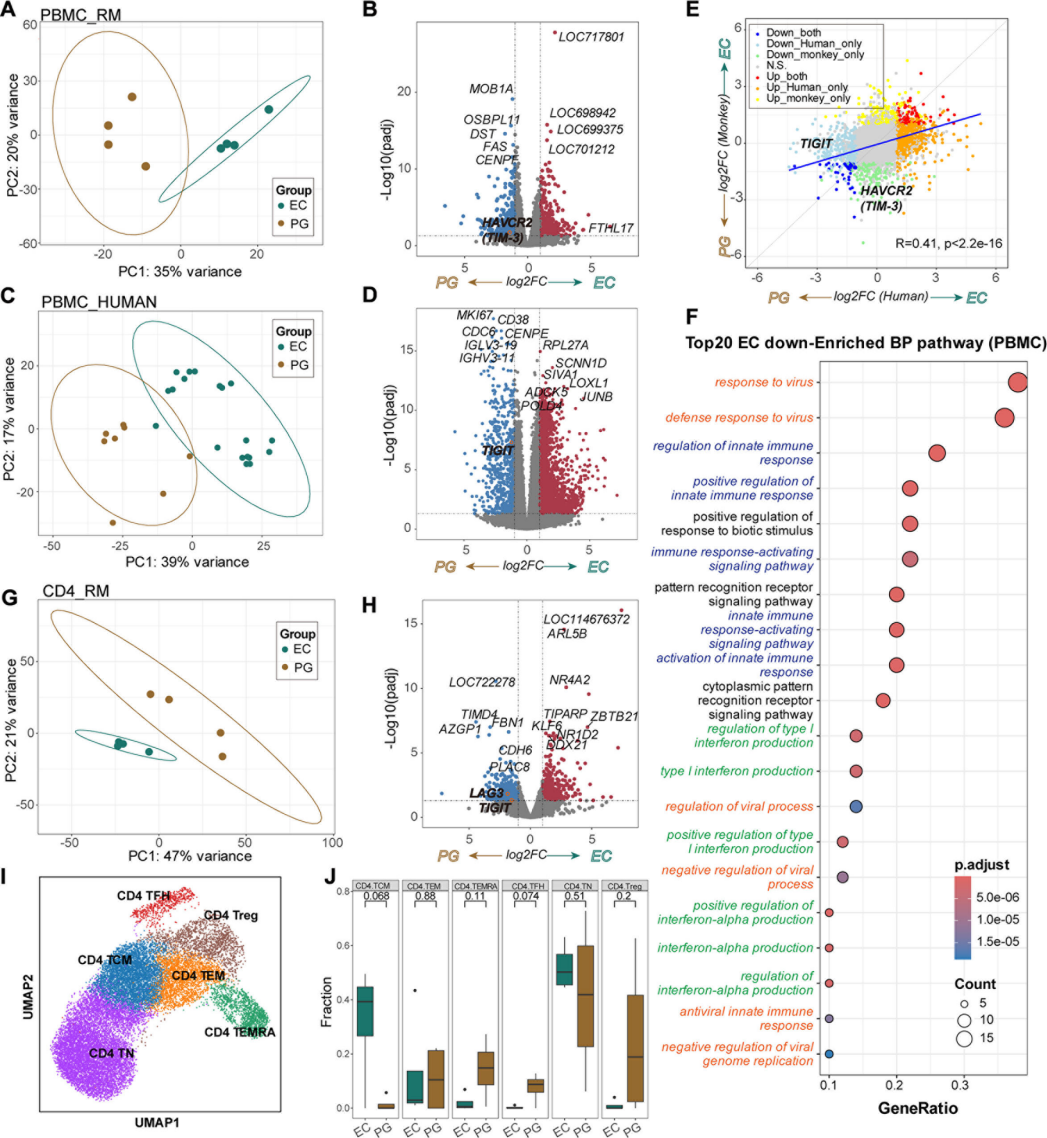
Transcriptomic analysis of PGs and ECs. (**A**) PCA of RNA-seq data from PBMCs of RMs PGs and ECs. (**B**) Volcano plot displaying differentially expressed genes from PBMCs of RMs ECs and PGs. (**C**) PCA of publicly available RNA-seq data from PBMCs of HIV-1-infected humans. (**D**) Volcano plot displaying differentially expressed genes from PBMCs of human ECs and PGs. (**E**) Scatter plot illustrating the relationship of log_2_ fold change (log_2_FC) of differentially expressed genes between PBMCs of infected humans and RMs. (**F**) Dot plot showing the Gene Ontology (GO) enrichment pathways of genes commonly downregulated in the EC groups of both humans and RMs, with immune response-related pathways highlighted in blue, virus response-related pathways highlighted in red, and interferon signaling-related pathways highlighted in green. (**G**) PCA of RNA-seq data from CD4^+^ T cells of RMs PGs and ECs. (**H**) Volcano plot showing differentially expressed genes between CD4^+^ T cells of RMs PGs and ECs. (**I**) UMAP visualization of CD4^+^ T-cell subtypes. (**J**) Boxplots displaying the proportions of CD4^+^ T-cell subtypes in the PG and EC groups based on deconvolution analysis of CD4^+^ T-cell RNA-seq data. *P*-values were calculated using the *t*-test.

We performed bulk RNA sequencing (RNA-seq) to profile PBMCs from ECs and PGs in RM models (Fig. 6A). Differential gene expression analysis of the RNA-seq data revealed significantly lower TIM-3 (encoded by *HAVCR2*) (|log2FC| > 1, adjusted *P*-value < 0.05) in ECs compared to PGs (Fig. 6B). To verify the translational relevance of our RM models and underscore its value in HIV research, we analyzed public RNA-seq data from PBMCs of HIV-1-infected patients, comprising 19 ECs and 8 PGs (Fig. 6C) (26). Consistent with RM data, TIM-3 (encoded by *HAVCR2*) expression was lower in human ECs with a similar magnitude (log2FC = −0.8, adjusted *P*-value = 1.67 × 10⁻⁵) (Table S1), while *TIGIT* was also suppressed in human ECs (Fig. 6D). Cross-species (human and RMs) correlation analysis ‌demonstrated‌ ‌significantly strong concordance‌ in differential gene expression profiles for PGs and ECs (R = 0.41, adjusted *P*-value＜2.2 × 10⁻^16^, Fig. 6E). In addition, Gene Ontology (GO) analysis of commonly downregulated genes in both humans and RMs in the EC groups revealed significant enrichment of viral response, interferon signaling, and immune response pathways (adjusted *P*-value < 0.05) (Fig. 6F).

Bulk RNA-seq analysis on RMs’ CD4^+^ T cells isolated from PBMCs of PGs and ECs (Fig. 6G) revealed significantly low levels of *TIGIT* and *LAG3* expression in the ECs (Fig. 6H), further confirming the above observation. Enrichment pathways of downregulated differentially expressed genes (DEGs) in the ECs, such as peptide metabolic and biosynthetic process pathways, suggested ECs may experience a milder immune response (Fig. S2A). To preliminarily understand the mechanisms linking low TIGIT expression in ECs associated with undetectable viral loads, we performed single-cell RNA sequencing (scRNA-seq) on magnetically sorted CD4^+^ T and CD8^+^ T cells from PBMCs of two RMs selected from a cohort of 14 RMs. One RM exhibited high TIGIT expression on CD8^+^ T cells and progressive high viremia following SHIV_SF62P3_ challenge, while the other showed low TIGIT expression on CD8^+^ T cells and sustained undetectable viral loads post-challenge (Fig. S3). After standard preprocessing of the scRNA-seq data from these two RMs, we excluded non-T cells and potential doublets (Fig. S2B to S2E). For CD4^+^ T cells from scRNA-seq, we identified six subsets: naive T (TN), central memory T (TCM), follicular helper T (TFH), effector memory T (TEM), terminally differentiated effector memory T (TEMRA), and regulatory T (Treg) cells (Fig. 6I). Notably, *TIGIT* was specifically expressed in TFH and Treg cells (Fig. S2F). Pseudo time analysis supports our CD4^+^ T-cell subtype annotations (Fig. S2G). Using the scRNA-seq data from CD4^+^ T cells, we applied BayesPrism to deconvolute the cellular proportions of CD4^+^ T-cell subtypes in the previous bulk RNA-seq data (Fig. 6J) and found ECs had higher proportions of CD4^+^ TCM cells, while PGs exhibited higher proportions of CD4^+^ TEMRA, CD4^+^ TFH, and CD4^+^ Treg cells. This implies that the bulk level of low-level TIGIT expression in ECs is likely due to the lower abundance of TIGIT^+^ CD4^+^-T cells in this group. We also identified six CD8^+^ T-cell subsets, including proliferating T (Tprof), TN, transitional follicular memory T (TFM), TEM, TEMRA, and mucosal-associated invariant T cells (MAIT) (Fig. S2H and S2I). Notably, CD8^+^ Tprof cells expressing activation markers *CD38* and *MAMU-DRA* also exhibited significantly elevated *TIGIT* expression (Fig. S2J).

### Low-level expression of TIGIT on CD8^+^ T cells is associated with an enhanced SIV-specific immune response in ECs

**Fig 7:**
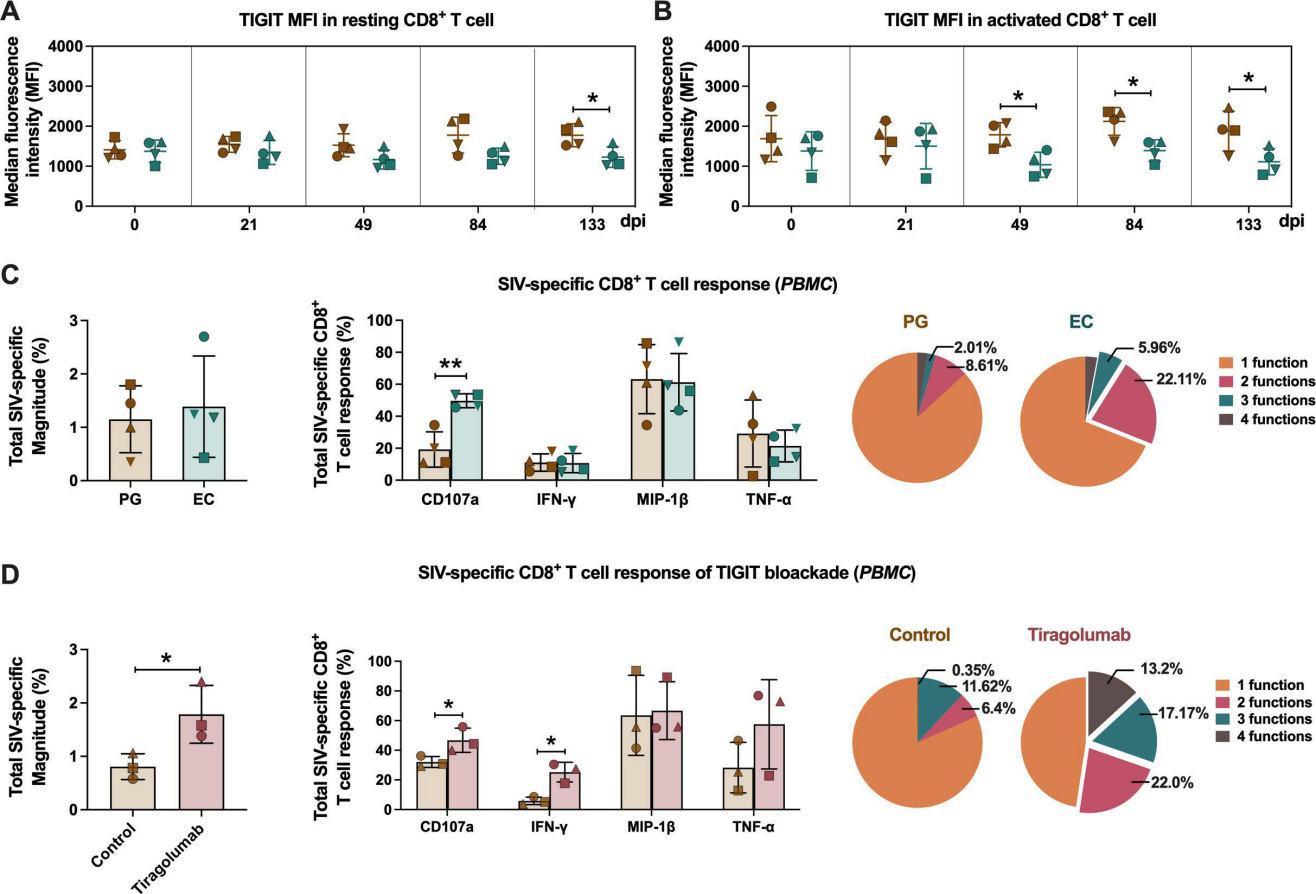
Lower expression of TIGIT and superior SIV-specific CD8^+^ T-cell functionality in ECs. (**A and B**) Comparison of the median fluorescence intensity (MFI) of TIGIT in resting CD8^+^ T cells (**A**) and activated CD8^+^ T cells (**B**) between PGs and ECs. (**C and D**) The total magnitude (left panels), quantification (middle panels), and polyfunctionality (right panels) of cytokine production from SIV-specific CD8^+^ T cells among PGs and ECs (133 dpi, **C**), PGs (133 dpi) treated with or without tiragolumab (10 µg/mL, **D**). The polyfunctionality was determined by a Boolean gate based on the frequencies of CD107a, IFN-γ, MIP-1β, and TNF-α from PBMCs. Statistics were performed by a two-tailed unpaired Student’s *t*-test between two groups at the indicated time points. Repeated measures ANOVA was applied to compare each group over all indicated time points. Error bars represent the mean ± SD. *P < 0.05.

Further analysis of TIGIT expression revealed differences between resting (HLA-DR^-^CD38^-^) CD8^+^ T cells and activated (HLA-DR^+^/CD38^+^) CD8^+^ T cells. We found that diminished TIGIT expression in CD8^+^ T cells of ECs was predominantly localized to activated CD8^+^ T cells during the acute to chronic infection phase (49, 84, and 133 days post-infection), whereas in resting CD8^+^ T cells, this reduction was only evident at 133 days post-infection (Fig. 7A and B).

The SIV Gag-specific CD8^+^ T-cell responses were evaluated, revealing that the proportions of CD8^+^ T cells producing CD107a were significantly higher in ECs, and polyfunctional SIV-specific CD8^+^ T cells (exhibiting two or three functional cytokines) were notably more pronounced in ECs compared to PGs (Fig. 7C), suggesting that the enhanced SIV-specific response is largely attributable to TIGIT expression. SIV-specific CD8^+^ T-cell frequencies were quantified after adjusting for background from negative controls (Fig. S4). Total response magnitude, as well as functional combinations of SIV-specific CD8^+^ T cells, was assessed via a Boolean gate strategy.

We also investigated the potential functional role of TIGIT on CD8^+^ T-cell immune response. TIGIT was found to interact with HIV-1 gp120 and share identical cellular localization (Fig. S5). Next, we employed the TIGIT blockade antibody (tiragolumab) to block the TIGIT pathway in CD8^+^ T cells from PGs during the chronic infection phase (133 days post-infection). The functionality of SIV-specific CD8^+^ T cells was assessed before and after treatment. TIGIT blockade significantly enhanced the magnitude of the SIV-specific CD8^+^ T-cell response, particularly increasing the expression of CD107a and IFN-γ. In addition, tiragolumab treatment promoted the emergence of double, triple, and quadruple functional SIV-specific CD8^+^ T-cell responses (Fig. 7D).

## DISCUSSION

Achieving an HIV functional cure is hampered by several factors, including but not limited to the long-term persistence of HIV reservoirs, T-cell dysfunction caused by chronic immune activation, and the lack of effective latency-reversing agents (LRAs). Nevertheless, fewer than 1% of people living with HIV without ART exhibit viral loads that fall below the detection threshold of commercial assays, referred to as ECs (2). The ECs have long served as a model for developing novel HIV functional cure strategies, underscoring the necessity of elucidating host immune factors that contribute to elite viral control.

Our longitudinal analysis revealed distinct temporal patterns of T-cell activation between EC and PG groups. Notably, CD4^+^ T cells in PGs exhibited earlier (day 49) and sustained elevation in HLA-DR^+^ and CD38^+^ expression compared to ECs, while significant differences in CD8^+^ T-cell activation only emerged on day 133. This temporal dissociation suggests that CD4^+^ T-cell activation may serve as an earlier immunological indicator of uncontrolled viral replication in this SHIV model, and CD8^+^ T cells demonstrate delayed or differentially regulated activation kinetics in response to persistent disease progression. The capacities of these activated T-cell subsets and their respective contributions to viral control or disease progression remain to be better understood in the future. These findings demonstrate the therapeutic rationale for ICB in HIV immunotherapy, as persistent immune activation drives T-cell exhaustion through sustained expression of checkpoint signals, thereby establishing their blockade as a critical therapeutic intervention. Numerous studies indicated that immune checkpoint blockade is beneficial for the reinvigoration of exhausted T cells and may serve as an HIV cure strategy by not only restoring HIV-specific immunity but also targeting latently infected cells (13). Due to the “test and treat” policy, it is harder for us to systematically investigate the potential role of ICs in long-term natural HIV disease progression without therapeutic intervention. Furthermore, reliance on easily accessible peripheral blood in most human studies may not fully interpret immune dynamics in tissues where critical virologic events occur.

Herein, we aim to deepen the understanding of IC dynamics by investigating their expression levels in the context of PGs and ECs using NHP models. Comparative analysis of the expression profiles of ICs in PBMCs and LNMCs revealed generally low-level expression of ICs, particularly BTLA in resting CD4^+^ T cells within PBMCs, TIGIT, and BTLA in resting CD4^+^ T cells and resting CD4^+^ TCM cells within both inguinal and mesenteric LNMCs of ECs. The differential IC patterns in peripheral blood and lymphoid tissues may directly underscore the necessity of multi-tissue evaluations to identify optimal therapeutic targets. Furthermore, through correlation analysis with viral DNA, we identified a positive association between the expression of TIGIT/BTLA and the viral reservoir, suggesting that low levels of these ICs may contribute to a reduction in viral reservoirs, thereby facilitating virological control. As reported, the sustained persistence of HIV-1 viral reservoirs within resting CD4^+^ T cells remains a major barrier to HIV-1 cure (27). Collectively, our findings further substantiate the role of TIGIT and BTLA in virological control and suggest their potential as targets for achieving a functional cure (15, 19, 28).

In addition, transcriptomic analysis between PGs and ECs revealed that CD4^+^ T cells from both groups exhibited downregulated DEGs enriched in pathways related to peptide metabolism and biosynthesis, indicating a relatively subdued immune activation profile in ECs (29). These conserved transcriptional signatures highlight cross-species convergence in metabolic dysregulation, with downregulated DEGs in metabolic pathways being enriched in ECs. This aligns with studies in humans and rhesus macaques showing that high-viremic individuals exhibit heightened metabolic alterations compared to low/aviremic controllers. Notably, the enrichment of downregulated DEGs in ECs reflects the low-level immune activation profile across species, suggesting that early intervention targeting these pathways could disrupt viral persistence and serve as a novel therapeutic strategy to limit disease progression (30, 31). Integrated analysis of scRNA-seq and bulk RNA-seq data indicated a higher proportion of CD4^+^ TCM in ECs, alongside increased frequencies of CD4^+^ Treg, TFH cells, and TEMRA cells in PGs. These findings suggest complete reconstitution of the immune system and long-term immune memory function in ECs, contrasting with more pronounced immunological suppression and a persistent viral immune response in PGs (32–34). According to RNA-seq data, TIGIT was significantly downregulated in CD4^+^ T cells, especially in CD4^+^ TFH and Treg cells, illustrating that the low expression of TIGIT in CD4^+^ T cells is closely associated with virological control (15). However, scRNA-seq analysis in this study, limited to biological replicates, precludes definitive conclusions about cell-type proportion differences due to potential inter-individual variability. Although we employed deconvolution algorithms to infer cell-type proportions from bulk RNA-seq samples and thereby partially mitigated the single-sample limitation, this method provides only computational evidence. Consequently, all mechanistic hypotheses derived from scRNA-seq data are preliminary and require validation in a larger, longitudinally sampled cohort.

In CD8^+^ T cells, a notably low level of ICs was also documented in ECs since the acute phase of infection. Prior research has demonstrated that TIGIT negatively regulates the functionality of HIV-specific CD8^+^ T cells, with TIGIT^+^ CD8^+^ T cells exhibiting a significantly lower production of cytokines (IL-2, IFN-γ, and TNF-α) compared to their TIGIT counterparts in individuals with chronic HIV infection (35). This study showed enhanced polyfunctional SIV-specific CD8^+^ T-cell responses in ECs, characterized by elevated CD107a production and dual/triple cytokine functionality, highlighting TIGIT expression as a critical modulator of antiviral efficacy.

At the end of our study, we explored the potential role of TIGIT and the *in vitro* efficacy of the TIGIT-blocking strategy in macaque models. TIGIT and HIV-1 gp120 interact and colocalize in cells, suggesting TIGIT may function via gp120 binding during SIV infection. TIGIT blockade elicited enhanced SIV-specific CD8^+^ T-cell response, particularly promoting multifunctional T-cell responses. Therefore, the higher expression of TIGIT on CD8^+^ T cells of PGs endorses the possibility that high levels of TIGIT predict the aggregation of inhibitory receptors, thereby diminishing the capacity of cytotoxic CD8^+^ T cells against virus replication in viremic monkeys compared to aviremic monkeys (35, 36). These results indicate that virus-specific CD8^+^ T immunity could be enhanced by TIGIT blockade, while future comprehensive *in vivo* evaluation using appropriate animal models with detailed immunological profiling is needed to confirm its therapeutic potential.

In summary, our preclinical studies in SHIV-infected NHP models are indispensable for understanding the mechanisms of virus control. We provided a comprehensive survey of the IC expression profiles in PGs and ECs, spanning from the healthy state prior to challenge, acute and chronic infection, to distinct clinical outcomes. These differential immune checkpoint expression patterns observed across disease stages and tissue compartments provide critical insights for designing optimized immunotherapy regimens. Future studies could explore tissue-specific delivery methods to target lymphoid reservoirs and combination regimens guided by individual ICs profiling, which may ultimately lead to functional cure strategies.

## MATERIALS AND METHODS

### Sample collection

In this comparative study, 47 RMs were recruited from our previously completed studies. These animals were confirmed to be free of infections caused by SIV, simian type D retrovirus, simian T-lymphotropic virus, and herpes B virus. The RMs underwent bi-weekly mucosal challenges with 500 TCID_50_ SHIV_SF162P3_ via rectal or vaginal routes over a 6-week period, viral loads and CD4^+^, CD8^+^ T cells were continuously monitored. RMs were tested for major histocompatibility complex class I (MHC-I) alleles Mamu-A*04, Mamu-A*08, Mamu-A*13, and Mamu-B*01, Mamu-B*04, Mamu-B*08, and Mamu-B*17.

Peripheral blood samples were collected from all macaques prior to the initial challenge, followed by twice a week collections for the first 6 weeks and weekly collections for the subsequent 9 weeks post-exposure. Monthly blood sampling continued for an additional two months. Inguinal and mesenteric lymph nodes (LNs) were harvested during necropsy (158 days post-challenge). PBMCs were isolated using Ficoll-density gradient centrifugation from the peripheral blood samples. LNMCs were obtained by homogenizing the lymph nodes in a digestive solution (PBS containing 5% penicillin/streptomycin, 0.21 U/mL Collagenase D, and 15 U/mL DNase I) and subsequently washing in PBS II (PBS with 2% FBS and 2 mM EDTA). Both PBMCs and LNMCs were cryopreserved in a freezing solution (90% FBS and 10% DMSO) and stored in liquid nitrogen for long-term preservation.

### MHC class I typing of rhesus macaques

Genomic DNA was extracted from PBMCs of rhesus macaques using the QIAamp DNA Mini Kit (QIAGEN, Valencia, CA, USA), according to the manufacturer’s instructions. MHC class I alleles (Mamu-A*04, -A*08, -A*13, -B*01, -B*04, -B*08, and -B*17) were genotyped through polymerase chain reaction with sequence-specific primers (PCR-SSP) using LA Taq DNA polymerase with GC buffers (TaKaRa, Kusatsu City, Shiga Prefecture, Japan). Amplification reactions were carried out in 25 µL reaction volumes under the following thermal cycling conditions: initial denaturation at 95°C for 3 minutes, 35 cycles of 95°C for 30 seconds, 60°C for 40 seconds, and 72°C for 40 seconds, with a final extension at 72°C for 6 minutes. The sequence-specific primer pairs are listed in Table 1. PCR products were separated by electrophoresis on 2% agarose gels in 1 × TAE buffer at a constant voltage.

**TABLE 1:** Sequence-specific primer pairs for typing MHC class I alleles

Primer	Primer sequence (5′−3′)
Mamu-A*04-for	TGT AAA ACG ACG GCC AGT CCA TGA GCT ATT TCT ACA CCT A
Mamu-A*04-rev	CAG GAA ACA GCT ATG ACC GGT AGG TTC TGT GCT GCT C
Mamu-A*08-for	TGT AAA ACG ACG GCC AGT CCT TGA GGT ATT TCT ACA CCG
Mamu-A*08-rev	CAG GAA ACA GCT ATG ACC GCA GCC ATG TCC GCT GCC
Mamu-A*13-for	TGT AAA ACG ACG GCC AGT ATG AGG TAT TTC TAC ACC TCC A
Mamu-A*13-rev	CAG GAA ACA GCT ATG ACC CCA GGT AGG CTC TCA TCC T
Mamu-B*01-for	TGT AAA ACG ACG GCC AGT ACC GGG AGA CAC GGA AGG
Mamu-B*01-rev	CAG GAA ACA GCT ATG ACC AGC CAC TCC ACG CAC CGG
Mamu-B*04-for	GCG CGA AAC GCC CAA AGA CAG
Mamu-B*04-rev	CTG GAC GCA GCC TGA GAG TAG
Mamu-B*08-for	CGT GAG GCG GAG CAG GTC
Mamu-B*08-rev	CCA CAG CTC CGA TGA ACA CAG
Mamu-B*17-for	TGT AAA ACG ACG GCC AGT GCG ACA CGG AGA GCC AAG GA
Mamu-B*17-rev	CAG GAA ACA GCT ATG ACC CCG CTC CGC ATA ACG GTT CC

### Quantification of viral RNA and DNA

Plasma viral RNA and proviral DNA from PBMCs or LNMCs were quantified using a qPCR assay. Initially, plasma viral RNA and proviral DNA were isolated utilizing the QIAmp Viral RNA Mini Kit and the DNeasy Blood & Tissue Kit, both produced by QIAGEN (Valencia, CA, USA). The primers designed to target SIV-gag91 are as follows: the forward primer is 5′-GCAGAGGAGGAAATTACCCAGTAC-3′ and the reverse primer is 5′-CAATTTTACCCAGGCATTTAATGTT-3′. The corresponding probe for SIV-gag91 is 5′-(FAM)-ACCTGCCATTAAGCCCGA-(MGB)−3′. The qPCR protocol consisted of an initial incubation at 48°C for 30 minutes, followed by a denaturation step at 95°C for 10 minutes, then 40 cycles of 95°C for 15 seconds, and a final extension at 60°C for 1 minute.

### SIV Gag peptide pool stimulation

Cryopreserved PBMCs were thawed and subsequently cultured in RPMI 1640 medium supplemented with 10% FBS and 2% penicillin/streptomycin at 37°C in 5% CO_2_ overnight. Following this incubation, the PBMCs were harvested and stimulated with SIV_mac239_ Gag peptides (obtained from NIH-ARP) at a final concentration of 2 µg/mL for each peptide. Control groups included untreated cells and those stimulated with a Cell Stimulation Cocktail (eBioscience, San Diego, CA, USA), which served as negative and positive controls, respectively. In the context of the TIGIT blockade experiment, PBMCs derived from PGs were treated with or without tiragolumab (an anti-TIGIT antibody from Selleck, at a final concentration of 10 µg/mL) during stimulation with SIV_mac239_ Gag peptides. Cells that were either not stimulated with SIV_mac239_ Gag peptides or stimulated with the Cell Stimulation Cocktail were again utilized as negative and positive controls. In addition, Brefeldin A (BioLegend, San Diego, CA, USA) and anti-CD107a-BV786 (BD Biosciences, San Jose, CA, USA) were introduced to all cell culture plates at the commencement of the experiment. All cells were maintained in culture for a duration of 6 hours at 37°C (37).

### Flow cytometry

To identify markers on PBMCs and LNMCs, a total of 1 × 10^6^ cells were resuspended in 100 µL of PBS and incubated with the Zombie NIR™ Fixable Viability Kit (BioLegend) at room temperature for 20 minutes to differentiate between live and dead cells. Subsequently, the cells were washed with Stain Buffer (BD Biosciences) and labeled with the appropriate extracellular antibodies at 4°C for 30 minutes. To assess intracellular cytokine production following stimulation with an SIV Gag peptide pool, the cells were fixed and permeabilized using the Fixation and Permeabilization Solution (BD Biosciences) and subsequently stained with anti-IFN-γ-BV711 (BioLegend), anti-MIP-1β-BV421 (BD Biosciences), and anti-TNF-α-BV650 (BioLegend) at 4°C for 30 minutes. The cells were then fixed in 1% paraformaldehyde and analyzed using BD LSR Fortessa within 24 hours. A comprehensive list of all antibodies utilized for flow cytometry is provided in Table 2. The identification of positive cell populations was determined using fluorescence-minus-one (FMO) controls, and the percentages of specific cell populations were reported relative to the parent populations.

**TABLE 2:** Antibodies for flow cytometry

Antigen	Fluorophore	Clone	Company	Catalog no.
CD3	PE	SP34-2	BD Biosciences	552127
CD3	BV605	SP34-2	BD Biosciences	562994
CD3	PerCP	SP34-2	BD Biosciences	552851
CD4	BV785	OKT4	BioLegend	317442
CD4	PE	OKT4	BioLegend	317410
CD8	PE	RPAT8	BD Biosciences	555367
CD8	BV510	RPA-T8	BioLegend	301048
CD8	FITC	RPA-T8	BD Biosciences	555366
CCR7	BV711	G043H7	BioLegend	353228
CCR7	BV421	G043H7	BioLegend	353208
CD45RA	PE-CF594	5H9	BD Biosciences	565419
CD45RA	APC	5H9	BD Biosciences	561210
PD-1	PerCP-Cy5.5	EH12.2H7	BioLegend	329914
CTLA-4	BV421	BNI3	BioLegend	369606
CD160	Alexa Fluor 488	BY55	BD Biosciences	562351
TIGIT	PE-Cy7	MBSA43	eBioscience	25-9500-42
BTLA	BV650	J168-540	BD Biosciences	564803
LAG-3	APC	polyclonal Ab	R&D Systems	FAB2319A
CD38	FITC	AT-1	Stemcell	60131FI
CD38	Alexa Fluor 700	AT-1	Novus Biologicals	NBP2-47908AF700
HLA-DR	BV510	G46-6	BD Biosciences	563083
HLA-DR	Alexa Fluor 700	G46-6	BD Biosciences	560743
TNF-α	BV650	MAb11	BioLegend	502938
MIP-1β	BV421	D21-1351	BD Biosciences	562900
IFN-γ	BV711	4S.B3	BioLegend	502540
CD107a	BV786	H4A3	BD Biosciences	563869

### Cell sorting for FACS

CD4^+^ and CD8^+^ T cells were immunomagnetic isolated from rhesus macaque peripheral blood mononuclear cells (PBMCs, <1 × 10^6^) using the EasySep PE Positive Selection Kit II (Stemcell Technologies, Vancouver, BC, Canada) following the manufacturer’s instructions. The PBMCs were stained with PE anti-human CD4 antibody (BioLegend) for CD4^+^ T cells or PE anti-human CD8 antibody (BD Biosciences) for CD8^+^ T cells. The stained cells were subsequently labeled with a PE selection cocktail and separated using an EasySep magnet by simply pouring off the unwanted cells. After separation, the purified CD4^+^ or CD8^+^ T cells were stained with PerCP anti-human CD3 antibody (BD Biosciences) to assess their purity using flow cytometry. The fluorescence was monitored by BD LSR Fortessa.

### RNA-seq processing and analysis

The RNA from PBMCs was extracted and sequenced by BGI Genomics Co., Ltd (Shenzhen, CN). Quality control was applied on raw paired fastq files of each sample using fastp (version 0.23.4) with default parameters (38). After quality control, fastq files of monkeys and humans were aligned to the *Rhesus Macaque* reference genome (Mmul_10) and GRCh38.p14 (Release 46) genome, respectively, using STAR (version 2.7.11b) with default parameters (39). Then, mapped reads were counted at the gene level using the FeatureCounts function of subread (version 2.0.6) to generate count matrices (40).

Differential gene expression analysis was conducted using DESeq2 (version 1.44.0) (41). Gene Ontology (GO) enrichment analysis was performed using clusterProfiler (version 4.12.6), and GO terms with adjusted *P*-value < 0.05 were defined as enriched pathways (42).

### scRNA-seq processing and analysis

The RNA from single CD4^+^ and CD8^+^ T cells was sequenced by OE Biotech Co., Ltd. (Shanghai, China). The Cell Ranger toolkit (version 8.0.1) provided by 10 × Genomics was applied to preprocess the fastq data, including filtering low-quality reads, aligning reads to Mmul_10, assigning cell barcodes, and generating the unique molecular identifier (UMI) matrix.

Scrublet was used to remove potential doublets (43). Then, the Python-based Scanpy toolkit (version 1.9.8) was employed to perform dimension reduction and two rounds of unsupervised clustering (44). For the first round of clustering, we annotated major cell types. Briefly, we filtered out genes detected in less than 10 cells and kept high-quality cells with a threshold of 500–4,000 genes. Then, the normalized expression matrix was calculated based on the raw UMI counts after normalizing total counts per cell (library size) and was then scaled by 1e4 and logarithmically transformed. Principal component analysis (PCA) was performed on the top 2,000 highly variable genes, and the top 30 components were used for downstream analyses. To correct the batch effects from different samples, we applied BBKNN to generate a batch-balanced k-nearest neighbor (KNN) graph. Then, the Uniform Manifold Approximation and Projection (UMAP) and Leiden algorithm were applied to such nearest neighbor graphs to visualize and detect communities and find cell clusters. After the first round of clustering and annotation, in addition to T cells (*CD3E*, *CD3D*), we also identified platelets (*PPBP*), pDCs (*CD79A*, *CD4*, *GZMB*, *CST3*, *LYZ*, *IL3RA*), plasma cells (*CD79A*, *JCHAIN*), and B cells (*CD79A*, *MS4A1*).

For the second round of clustering, we first excluded non-T cells, low-quality T cells, and doublets, and samples of CD4^+^ and CD8^+^ T cells underwent the same workflow separately to identify CD4^+^ and CD8^+^ T-cell subtypes. Specifically, we filtered genes expressed in less than 5 cells and used the harmonypy algorithm to adjust the principal components, while other steps were the same as the first round of clustering (45).

Monocle3 (version 1.3.7) was applied to construct single-cell trajectories and predict the pseudotime of each cell, with a starting node set within TN subtypes for both CD4^+^ and CD8^+^ T cells (46). BayesPrism (version 2.2.2) was utilized to perform cell-type deconvolution analysis to predict the proportion of each CD4^+^ T-cell subtype within bulk CD4^+^ RNA-seq data based on raw count matrices (47).

### Immunoprecipitation and immunoblot analysis

HEK 293T cells (obtained from ATCC) were collected 48 hours post-transfection. Cell lysates were prepared in lysis buffer containing 1% Nonidet P-40 and protease inhibitor cocktail (Roche, Basel, Switzerland). Subsequently, soluble proteins were immunoprecipitated using anti-Flag (M2, Sigma, St. Louis, MO, USA). An aliquot of the total lysate (5%, vol/vol) was included as a control. Immunoblotting was performed with horseradish peroxidase (HRP)-conjugated anti-HA (Cell Signaling Technology, Danvers, MA, USA), HRP-conjugated anti-Flag (Sigma), and HRP-conjugated anti-β-actin (Abcam, Cambridge, UK). The antigen-antibody complexes were visualized via chemiluminescence (Immobilon Western Chemiluminescent HRP Substrate, Millipore, Billerica, MA, USA). A PageRuler Western marker (Thermo, Waltham, MA, USA) was used as a molecular weight standard.

### Immunofluorescence

HeLa cells plated on glass coverslips were transfected with the indicated plasmids and collected 48 hours post-transfection. After fixation with 4% paraformaldehyde (PFA) for 15 min, the cells were permeabilized with 0.3% Triton X-100 in PBS for 15 minutes. A blocking buffer was added to each slide for 30 minutes at 37°C. Mouse anti-Flag (1:100) and rabbit anti-HA antibodies were incubated at 4°C overnight. The cells were washed three times with PBST buffer and then incubated with FITC-conjugated goat anti-mouse IgG (1:100) and TRITC-conjugated goat anti-rabbit IgG (1:100) secondary antibodies (ZSGB-Bio, Beijing, CN) were incubated at room temperature for another hour. The cells were then stained with DAPI and imaged by a laser scanning confocal microscope with a 63× oil immersion lens.

### Statistical analysis

All data were analyzed utilizing GraphPad Prism Version 9.2.0. The results are expressed as the mean ± standard deviation (SD), and statistical comparisons between the two groups were conducted using either a two-tailed unpaired Student’s *t*-test or Welch’s *t*-test. Repeated measures ANOVA was used to compare each group over all indicated time points. In addition, Spearman rank correlation analysis was employed to evaluate the relationships among various indicators. Statistically significant results are denoted by one to three asterisks, with significance levels defined as *P < 0.05, **P < 0.01, and ***P < 0.001.

## Data Availability

Sequencing data are available in Genome Sequence Archive (RNA seq and scRNA-seq, CRA025372). The code for RNA sequencing analysis is available from the corresponding author upon reasonable request.

## SUPPLEMENTAL MATERIAL

Supplemental figures jvi.00785-25-s0001.docx Fig. S1 to S5.

Table S1 jvi.00785-25-s0002.xlsx Differentially expressed genes of humans and rhesus macaques in PBMC.

ASM does not own the copyrights to Supplemental Material that may be linked to, or accessed through, an article. The authors have granted ASM a non-exclusive, world-wide license to publish the Supplemental Material files. Please contact the corresponding author directly for reuse.
