# Supplementary material for: Novel insights into immune checkpoints in HIV/SHIV infection: from SHIVSF162P3-infected elite controllers to therapeutic strategy
Source: J Virol. 2025 Jul 10;99(8):e00785-25. doi: 10.1128/jvi.00785-25 (PMC12363197; doi:10.1128/jvi.00785-25)
Supplement: Supplemental figures — Fig. S1 to S5. [file jvi.00785-25-s0001.docx]

**Supplemental Material**

**Novel insights into immune checkpoints in HIV/SHIV infection: from SHIV_SF162P3_-infected elite controllers to therapeutic strategy**

**Yuting Sun,^1,#^ Chenbo Yang,^1,#^ Peiwen Liu,^2,#^ Zhe Cong,^1,3,4^ Jiahui Luo,^1^ Ling Tong,^1^ Jingjing Zhang,^1,3^ Jiahan Lu,^1,3^ Ziqing Jia,^1^ Lin Zhu,^1,3^ Qiuhan Lu,^1^ Ting Chen,^1,3^ Qiang Wei,^1,3,4,5^ Dan Li^6,^*, Rui Hou^7,^* Jing Xue^1,3,4,5,^***

**AUTHOR AFFILIATIONS**

^1^ NHC Key Laboratory of Human Disease Comparative Medicine, Beijing Key Laboratory for Animal Models of Emerging and Remerging Infectious Diseases, Institute of Laboratory Animal Science, Chinese Academy of Medical Sciences and Peking Union Medical College, Beijing, China

^2^ Harry Perkins Institute of Medical Research, QEII Medical Centre and Centre for Medical Research, the University of Western Australia, Perth, Western Australia, Australia

^3^ National Center of Technology Innovation for animal model, National Human Diseases Animal Model Resource Center, Institute of Laboratory Animal Science, Chinese Academy of Medical Sciences, Beijing, China

^4^ Center for AIDS Research, Chinese Academy of Medical Sciences and Peking Union Medical College, Beijing, China

^5^ State Key Laboratory of Respiratory Health and Multimorbidity, Key Laboratory of Pathogen Infection Prevention and Control (Peking Union Medical College), Ministry of Education, Institute of Laboratory Animal Science, Chinese Academy of Medical Sciences, Beijing, China

^6^ National Key Laboratory of Intelligent Tracking and Forecasting for Infectious Diseases, National Center for AIDS/ STD Control and Prevention, Chinese Center for Disease Control and Prevention, Beijing, China

^7^ Shenzhen Clinical Research Centre for Geriatrics, Shenzhen People’s Hospital (The Second Clinical Medical College, Jinan University, The First Affiliated Hospital, Southern University of Science and Technology) Shenzhen, Guangdong, China

^#^ These authors contributed equally

*Correspondence to: [lidan@chinaaids](mailto:lidan@chinaaids).cn (D.L.), asrhou@gmail.com (R.H.), [xuejing@cnilas.org](mailto:xuejing@cnilas.org) (J.X.)

This PDF file includes:

Fig S1 to S5.

**
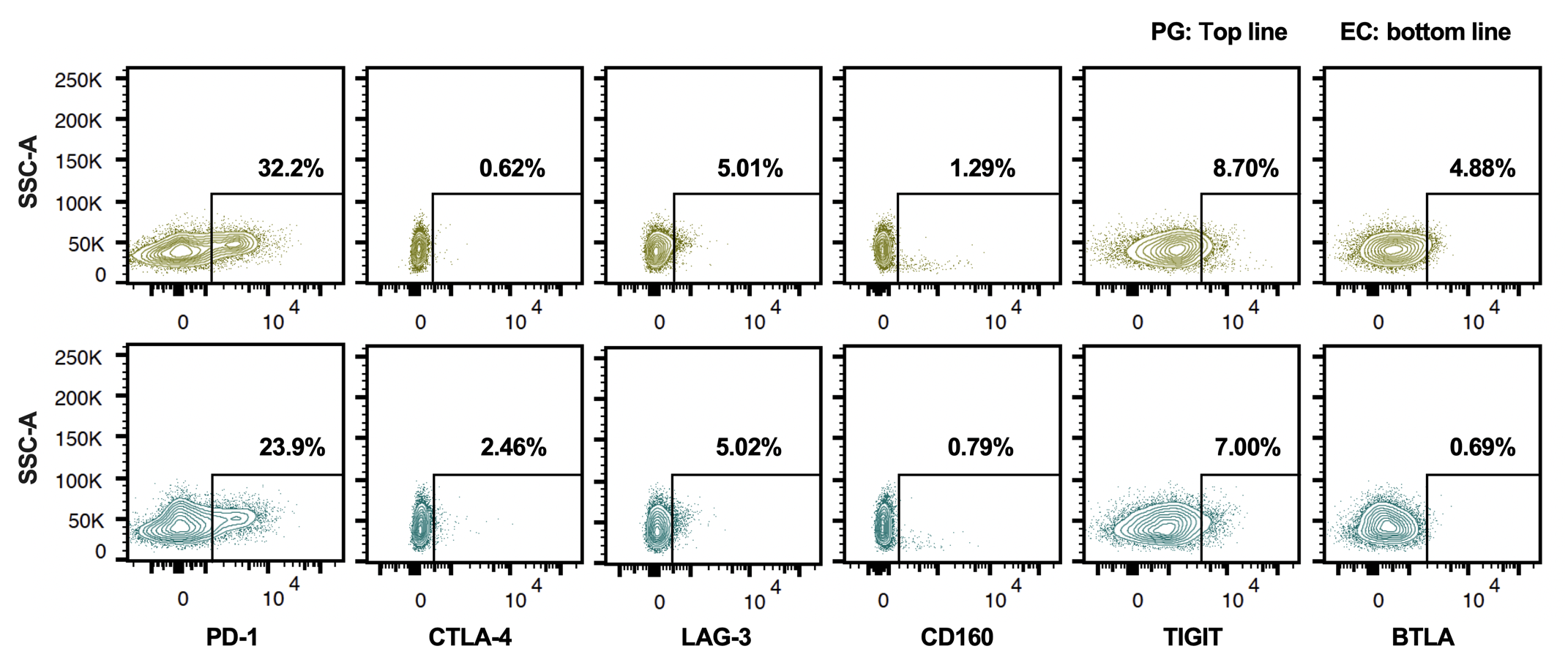
**

**Fig S1** Representative flow plots of ICs on CD4^+^ T cells at 49 days post infection. Gating schemes of PD-1, CTLA-4, LAG-3, CD160, TIGIT and BTLA were shown.


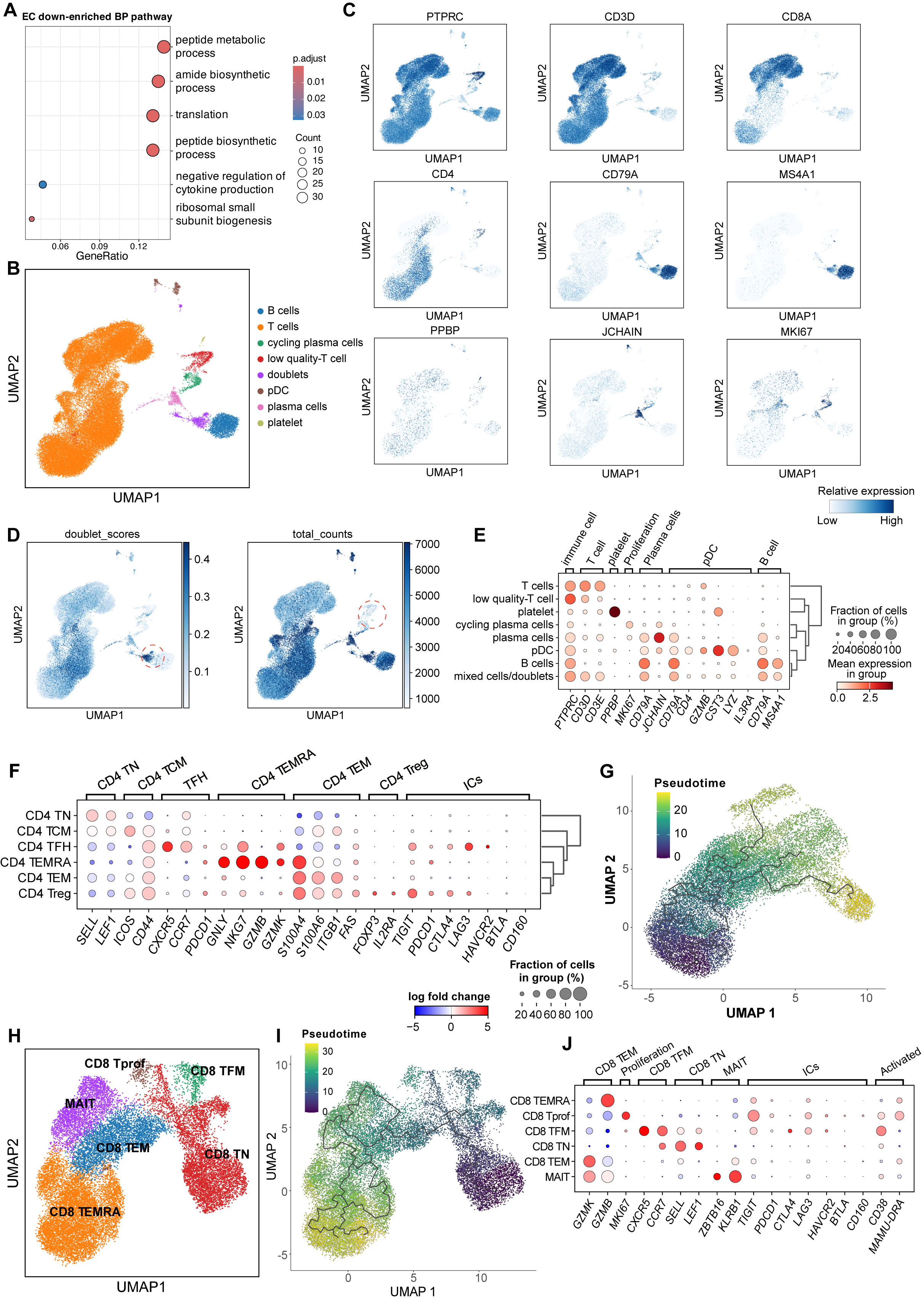


**Fig S2** Transcriptomic analysis of PGs and ECs. (A) Dot plot showing the enriched GO pathways of down-regulated genes in the CD4^+^ T cells from ECs. (B) UMAP visualization of major cell types across all samples from PGs and ECs. (C) UMAP visualization of the expression of selected marker genes in major cell types from PGs and ECs. (D) UMAP visualization displaying doublet scores and total counts for each cell across PGs and ECs. (E) Dot plot showing the expression patterns of marker genes across distinct major cell types from PGs and ECs. (F) Dot plot showing the expression patterns of marker genes across distinct CD4^+^ T cell subtypes from PGs and ECs. (G) Pseudo time analysis beginning with naïve CD4^+^ T cells (CD4^+^ TN). Colors from dark blue to yellow represent less differentiated to more differentiated cells. (H) UMAP visualization of CD8^+^ T-cell subtypes (I) Pseudo time analysis beginning with naïve CD8^+^ T cells (CD8^+^ TN). Colors from dark blue to yellow represent less differentiated to more differentiated cells. (J) Dot plot showing the expression patterns of marker genes across distinct CD8^+^ T-cell subtypes from PGs and ECs.


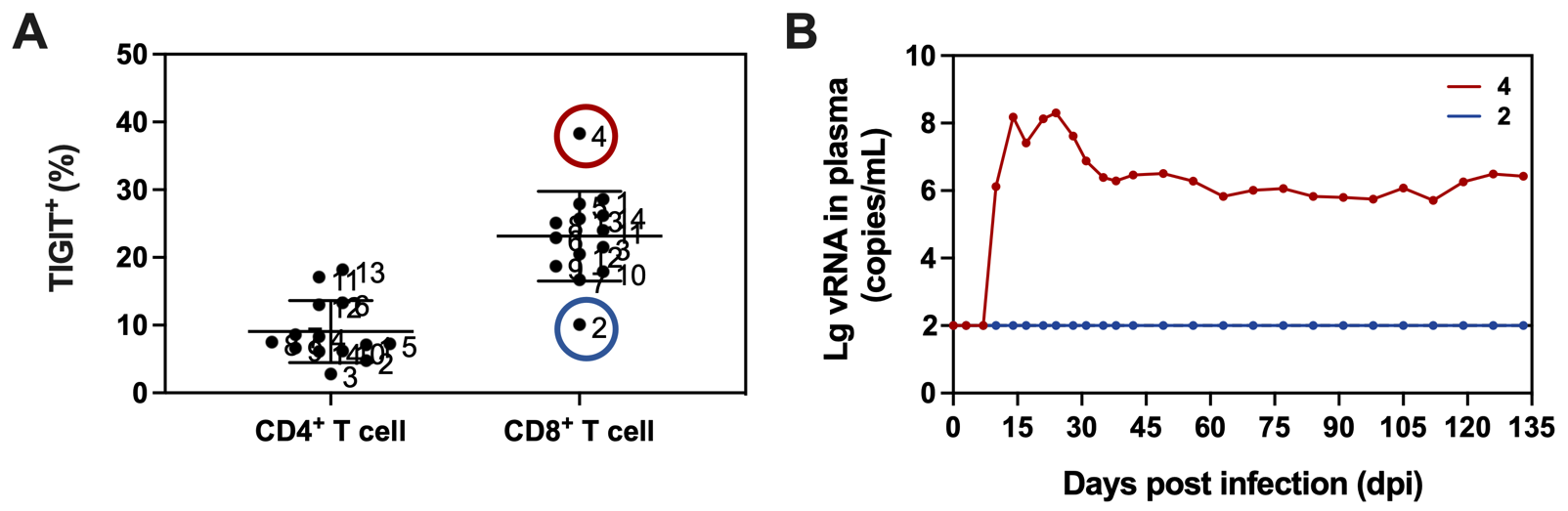


**Fig S3** TIGIT expression and vial loads in SHIV_SF162P3_-challenged RMs. (A) Frequencies of TIGIT^+^ cells among CD4^+^ and CD8^+^ T cells from PBMCs of 14 RMs at 18 weeks post-SHIV_SF162P3_ mucosal challenge, measured by flow cytometry. (B) Plasma viral loads in two RMs monitored for 135 days post-SHIV_SF162P3_ mucosal challenge. One with high TIGIT expression on CD8^+^ T cells and progressive viremia (red line), and one with low TIGIT expression on CD8^+^ T cells and sustained undetectable viral loads (blue line).


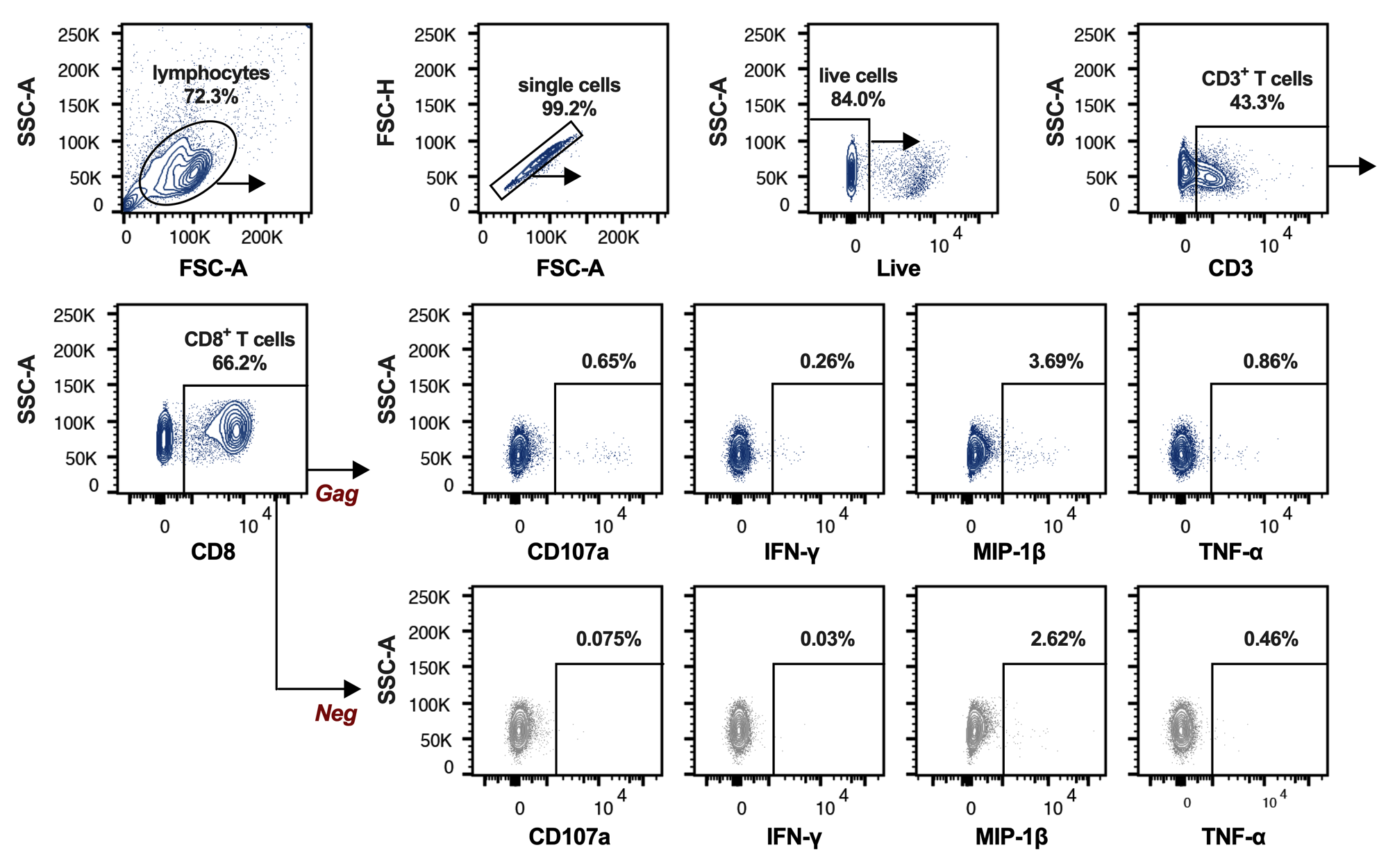


**Fig S4** Representative flow plots of SIV-specific functional cytokines on CD8^+^ T cell. Gating schemes of CD107a, IFN-γ, MIP-1β and TNF-α on CD8^+^ T cells stimulated with or without SIVmac239 Gag peptides from PBMCs.


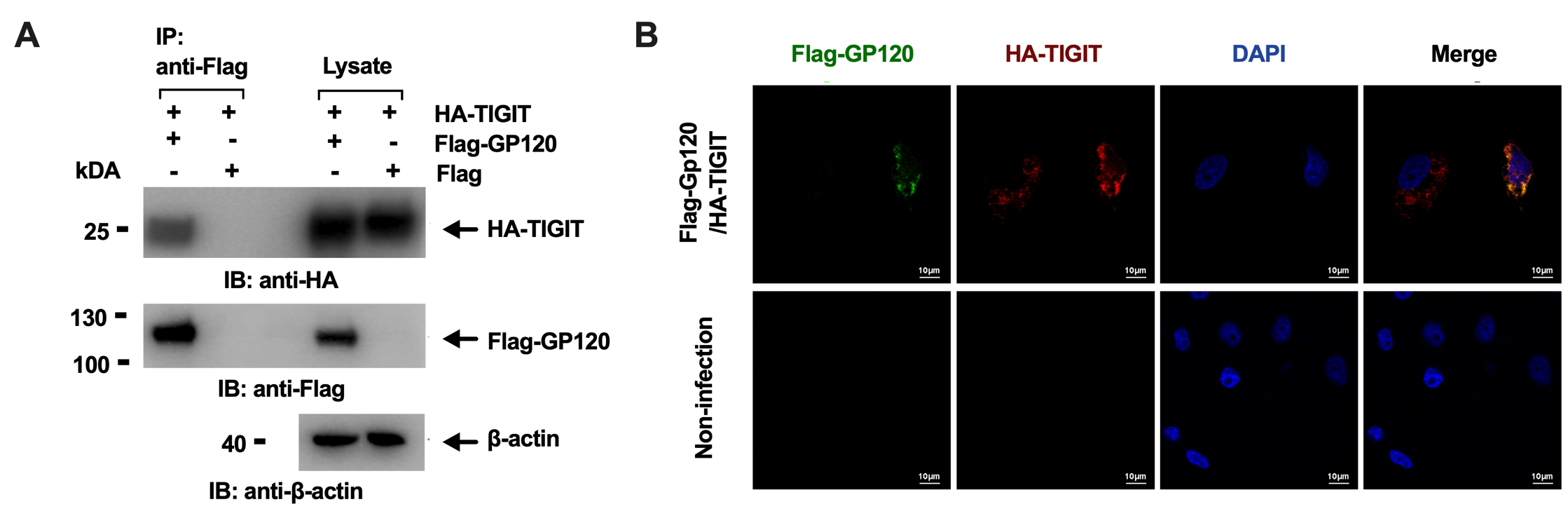


**Fig S5** The interaction and co-localization of TIGIT with gp120. (A) HEK293 cells lysates transfected with the indicated plasmids were subjected to anti-Flag immunoprecipitation and analyzed by immunoblotting. (B) HeLa cells were co-transfected with/without Flag-GP120 and HA-TIGIT and immune-stained with anti-Flag (green) and anti-HA (red) antibodies. Nuclei were stained with DAPI (blue). At least two independent repeats were performed in these experiments.
